# Efficient Adsorption of Pb(II) from Aqueous Solutions by Metal Organic Framework (Zn-BDC) Coated Magnetic Montmorillonite

**DOI:** 10.3390/polym10121383

**Published:** 2018-12-13

**Authors:** Jian Shen, Nan Wang, Yang Guang Wang, Di Yu, Xiao–kun Ouyang

**Affiliations:** 1 School of Materials and Chemical Engineering, Ningbo University of Technology, Ningbo 315211, China; shenjian0197@163.com; 2 School of Food and Pharmacy, Zhejiang Ocean University, Zhoushan 316022, China; wangyg0510@163.com (Y.G.W.); oyxk1973@163.com (D.Y.)

**Keywords:** Zn-BDC, magnetic montmorillonite, Pb(II), adsorption

## Abstract

Composite adsorption materials combine the advantages of various adsorptive materials and compensate for the defects of single adsorbents. Magnetic montmorillonite (MMMT) shows good adsorption properties for Pb(II). In order to further improve the adsorption properties of MMMT, in this work, Zn-BDC, a kind of metal–organic framework (MOF), was modified onto the surface of MMMT by in situ polymerization. The composite material MMMT@Zn-BDC was characterized by Zetasizer, SEM, TEM, FTIR, XRD, VSM, and XPS. The influence of adsorption conditions on the adsorption capacity of MMMT@Zn-BDC for Pb(II) was examined, including the adsorbent dosage, pH of Pb(II) solution, initial concentration of Pb(II), and the temperature and adsorption time. Also, the adsorption mechanism was studied. The results of this study show that MMMT@Zn-BDC adsorbs Pb(II) via chemisorption. In addition, MMMT@Zn-BDC exhibits good potential for adsorbing Pb(II), including its high adsorption capacity (724.64 mg/g) and good recyclability.

## 1. Introduction

Heavy metal pollution in water is becoming a more serious problem with increasing industrialization. Heavy metal pollution is a serious threat to the human body, ecological environment, and economic development [1,2]. Heavy metals are highly toxic, are easily enriched in organisms, and cannot be degraded naturally [3]. Trace heavy metals cause serious harm to microorganisms, aquatic plants, and animals. They can also enter the human body through the food chain or drinking water and cause serious injury and disease [4,5]. Lead is one such heavy metal. Lead and its compounds are frequently used as raw materials for modern industrial transportation, they are also used in metallurgy, printing, military and electronics. Incidents of excessive Pb(II) in liquid food and water have been reported many times [6,7]. Lead poisoning is a kind of accumulative poisoning. With the increasing accumulation of lead in the human body, people have a higher risk of kidney damage, hematopoietic dysfunction, and mental impairment [8]. 

Heavy metal wastewater is usually treated by ion exchange, biological treatment, precipitation, membrane permeation and adsorption [9,10]. The adsorption method [11,12] is widely used because of its performance efficiency, simple operation, selectivity, lack of pollution and low cost. Magnetic adsorbent materials represent a new type of adsorbent and have been shown to effectively adsorb lead ions [13,14]. More importantly, applying a magnetic field can easily separate the adsorbent from the water, which is beneficial for reusing the materials and concentrating heavy metals during treatment. Magnetic particles, especially Fe_3_O_4_, are easy to prepare and inexpensive [15,16,17]. However, Fe_3_O_4_ is easy to collect using the magnetic dipole effect [18]. Montmorillonite (MMT) is a lamellar crystalline mineral, consisting of eight layers sandwiched between two layers of tetrahedra. The cations in the middle of the interlayer include K^+^, Na^+^, Ca^2+^, Mg^2+^ and so on, which balance the negative charges of the main layers [19,20]. These cations can be exchanged by other cations with a high valence state and concentration [21]. In addition, the surface of MMT is electronegative; thus, it easily electrostatically adsorbs heavy metal ions such as lead ions [22]. Therefore, MMT is an ideal adsorbent for heavy metals [16,23,24]. However, as a dispersible adsorbent, MMT is not easy to recover; in contrast, magnetic MMT (MMMT) is both easily separated and exhibits good adsorption properties.

During this study, we found that MMMT has unsatisfactory adsorption capacity when the is Pb(II) concentration is high. Therefore, we considered modifying MMMT so that it can cope effectively with both high and low concentrations of Pb(II). Metal–organic framework (MOF) materials were first proposed by the Yaghi group [25]. MOFs are zeolite materials with a network structure, and they can be self-assembled from organic compounds and metal ions [26]. The pore size ratio and specific surface area of MOFs is high [27]. The physical and chemical properties of MOF materials are stable, and they can also be easily recycled. These advantages make MOFs an excellent adsorbent [28,29,30]. Especially in the field of heavy metal adsorption, MOFs have shown excellent adsorption properties [31,32]. Zn-BDC is a type of MOF that can be prepared from Zn^2+^ and terephthalic acid and catalyzed by triethylamine at room temperature. In this study, Zn-BDC was coated onto the surface of MMMT via in situ polymerization, thus obtaining the composite MMMT@Zn-BDC.

In this study, we propose a simple in situ polymerization method to combine MMMT with Zn-BDC. This composite material not only exhibits better recyclability but can also cope with a higher concentration of Pb(II). Additionally, it has good potential for the removal of Pb(II) from water and liquid food.

## 2. Materials and Methods 

### 2.1. Materials 

Pb(NO_3_)_2_, Zn(CH_3_CO_2_)_2_·6H_2_O, 1,4-dicarboxybenzene (BDC), FeCl_3_·6H_2_O, FeCl_2_·4H_2_O, MMT (>98%) were purchased from Aladdin Chemical Reagent Co., Ltd. (Shanghai, China). Methanol, triethylamine, *N,N*-dimethylformamide (DMF), and ammonia were all analytical reagent grade.

### 2.2. Preparation of MMMT@Zn-BDC

#### 2.2.1. Preparation of Zn-BDC

The preparation of Zn-BDC was done according to the method in [33]. The procedure was as follows: Zn(CH_3_CO)_2_ (1.699 g) and BDC (0.506 g) were separately dissolved in 200 mL and 100mL of DMF. These two solutions were combined and stirred for 30 min. Then, 0.85 mL of TEA was slowly dropped into the mixed solution and stirred for 2 h at room temperature. The white precipitates produced were collected by centrifugation. The product was washed alternately with DMF and methanol. Then, the product was immersed in methanol, which was replaced every 12 h to remove the DMF in Zn-BDC. After repeating the centrifugation, washing, and immersion steps two times, the product was dried for 6 h at 60 °C under vacuum condition. The final mass of Zn-BDC was 1 g. 

#### 2.2.2. Preparation of MMMT

The co-precipitation method was applied to synthesize MMMT [34]. Various amounts of MMT (0.5, 1, 1.5, or 2 g) were separately dispersed in 70 mL of deionized water; then, the dispersions were transferred into round-bottomed flasks. At the same time, nitrogen was continuously flowed into the dispersions to remove oxygen from the dispersions. After 30 min, FeCl_3_·6H_2_O (0.01 mol) and FeCl_2_·4H_2_O (0.005 mol) were dissolved in these dispersions, the reaction temperature was 70 °C, and the pH was adjusted to 9–10 using ammonia. After stirring for 30 min, the products were separated by external magnetic field. Next, the separated MMMT products were washed with alternating deionized water and ethanol, and finally, the product was dried in a freeze-drying box. Using this same method but without adding MMT, the resulting weight of Fe_3_O_4_ would be 1 g.

#### 2.2.3. Preparation of MMMT@Zn-BDC

**Figure 1:**
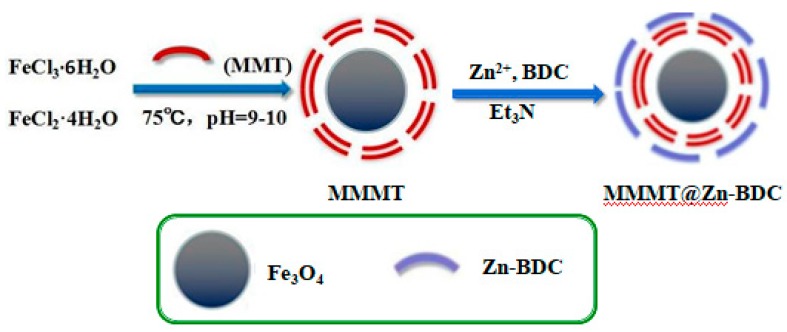
Synthesis of MMMT@Zn-BDC.

First, MMMT (1 g) was added to a round-bottomed flask containing 33 mL of DMF. Then, Zn(CH_3_CO)_2_ (1.699 g) was dissolved in this dispersion, and BDC (0.506 g) was separately dissolved in 33 mL of DMF. After stirring for 1 h, the dispersion and BDC solution were mixed. TEA (0.85 mL) was slowly added to the resulting suspension; then, after continuously stirring for 2 h, the reaction was stopped. This synthesis process is shown schematically in Figure 1. The products were separated and washed using the same process as used in the preparation of Zn-BDC, and finally, they were dried in a freeze-drying box.

### 2.3. Characterization

The surface morphology was recorded by scanning electron microscopy (SEM, S4800, Hitachi, Tokyo, Japan). A transmission electron microscope (TEM, JEM-2100, Lorentz, Tokyo, Japan) and energy dispersive spectrometer (EDS, GENESIS, EDAX, Mahwah, NJ, USA) were used to analyze the internal structures and composition of the materials. Nitrogen adsorption isotherms were obtained using Micromeritics ASAP 2020M. Functional groups of the adsorbent were determined using a Fourier transform infrared spectrometer (FTIR, Bruker, Karlsruhe, Germany). The crystallinity was determined by X-ray diffraction (XRD, Bruker, Karlsruhe, Germany). The magnetic properties were evaluated by vibrating sample magnetometry (VSM) analysis (SQUID-VSM, Quantum Design, Santiago, CA, USA). The elements of the adsorbent were analyzed by X-ray photoelectron spectroscopy (XPS, AXIS ULTRA DLD, Shimadzu, Tokyo, Japan).

### 2.4. Adsorption Experiment

The mother Pb(II) solution was prepared by Pb(NO_3_)_2_, and the resulting Pb(II) concentration was 1000 mg/L. Various Pb(II) solutions were diluted from this mother liquor. A Pb(II) solution (20 mL, 200 mg/L) was placed into several flasks, and various dosages of MMMT@Zn-BDC from 5 to 20 mg were separately added to these flasks. HNO_3_ and NaOH (0.1 mol/L) were applied to adjust the pH of the Pb(II) solution from 2 to 6. Then, the flasks were placed in an oscillator; the speed of revolution was 150 rpm. After the adsorption finished, the adsorbent was recovered using magnetic field. The concentration of residual Pb(II) solution was detected with an atomic absorbance spectrophotometer. The adsorption capacity of MMMT@Zn-BDC for Pb(II) at a certain time (*q*_t_) was calculated by Equation (1), Equation (2) was used to calculate the adsorption capacity of MMMT@Zn-BDC for Pb(II) to be in equilibrium (*q*_e_).

(1) $$q_{t}=\frac{(C_{0}-C_{t})V}{m}$$

(2) $$q_{e}=\frac{(C_{0}-C_{e})V}{m}$$

*C*_0_ (mg/L) is the initial concentration of Pb(II), *C*_t_ (mg/L) is the concentration of Pb(II) at a certain time during the process of adsorption, *V* (mL) is the volume of the Pb(II) solution, *m* (mg) is the dosage of adsorbent. *C*_e_ is the remaining Pb(II) concentration after the adsorption process reaching equilibrium. Finally, Equation (3) shows the calculated method of removal ratio (*R*) of MMMT@Zn-BDC for Pb(II):

(3) $$R=\frac{C_{0}-C_{e}}{C_{0}}\times 100\%$$

### 2.5. Reutilization Test

HCl (0.1 mol/L) was used to elute Pb(II) loaded in MMMT@Zn-BDC. The elution process was as follows: Pb(II)-loaded MMMT@Zn-BDC and the eluent were added into a conical flask, and the concentration was maintained at 1 g/L. Then, the flask was placed in the oscillator, the eluent was replaced every 3 h until Pb(II) could no longer be detected in the eluent. After elution, the adsorbent was freeze-dried and then reused four times to evaluate the relationship between reuse times and the adsorption capacity. 

## 3. Results and Discussion

### 3.1. Optimize the Preparation Conditions of MMMT@Zn-BDC

We studied the effects of adding MMT and Zn-BDC on the adsorption capacity and the binding rate (*BR*) of MMT and Zn-BDC coated onto the adsorbent to the actual weight added in the reaction solution. The results are shown in Table 1.

(4) $$BR=\frac{m}{M}\times 100\%$$

where *m* (g) is the mass of MMT and Zn-BDC loaded onto the adsorbent, and *M* (g) is the weight of MMT and Zn-BDC inserted in the reaction solution. 

According to Table 1, when the mass ratio of MMT to Fe_3_O_4_ is 1:1, the *BR* and the adsorption capacity showed a good result. Based on the optimal ratio (1:1) of MMT to Fe_3_O_4_, we further optimized the amount of added Zn-BDC. Considering both *BR* and the adsorption capacity, we chose 1:1 as the reaction mass ratio of MMMT and Zn-BDC.

### 3.2. Characterization

#### 3.2.1. SEM Observation

**Figure 2:**
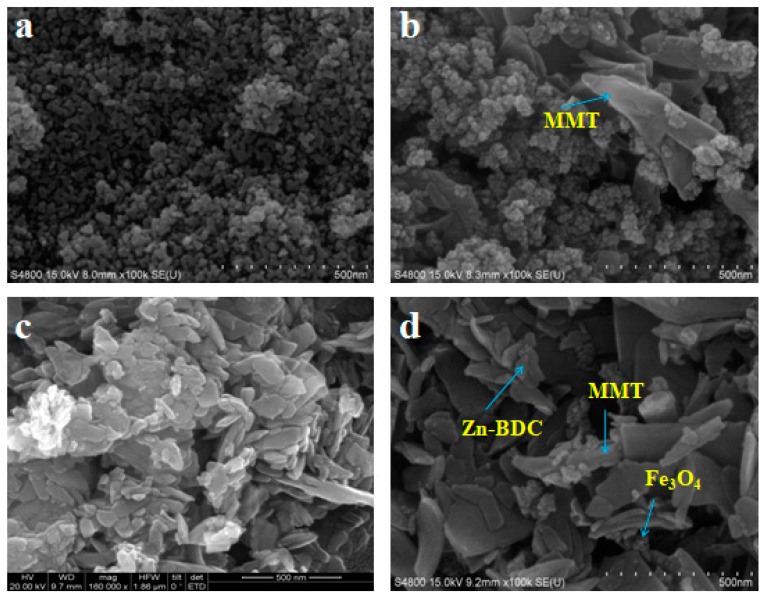
SEM images of (**a**) Fe_3_O_4_, (**b**) MMMT, (**c**) Zn-BDC, and (**d**) MMMT@Zn-BDC.

SEM images of Fe_3_O_4_, MMMT, Zn-BDC, and MMMT@Zn-BDC are shown in Figure 2. In Figure 2a, the morphology of magnetic nanoparticles is agglomerated spherical. After MMT was coated onto Fe_3_O_4_ by co-precipitation method, as shown in Figure 2b, irregular sheet MMT adheres to surface of Fe_3_O_4_. In Figure 2c, Zn-BDC shows an uneven crystal shape and size because it was prepared at room temperature [35]. In Figure 2d, after Zn-BDC was coated onto MMMT via in situ polymerization, short, rod-shaped crystals of Zn-BDC appear on the rough surface of the MMMT. The introduction of MMMT changed the size of the Zn-BDC crystal.

#### 3.2.2. TEM Analysis 

**Figure 3:**
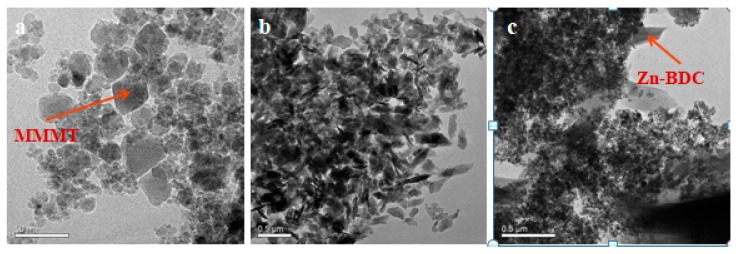
TEM of (**a**) MMMT, (**b**) Zn-BDC, and (**c**) MMMT@Zn-BDC.

TEM was used to analyze the internal structures and composition of MMMT, Zn-BDC, and MMMT@Zn-BDC. As shown in Figure 3a, when the amplification size is 500 nm, Fe_3_O_4_ particles are nearly spherical, while the irregular crystals are MMT. Fe_3_O_4_ and MMT are distributed almost in the same plane. It was confirmed that MMMT had been prepared successfully. In Figure 3b, the Zn-BDC crystals are also irregular, and it shows a slender lamellar structure. After synthesizing Zn-BDC on the MMMT surface, a slender lamellar structure appeared as can be seen in Figure 3c. It was concluded that a slender lamellar structure had been prepared successfully.

#### 3.2.3. N_2_ Adsorption and Desorption Analysis

**Figure 4:**
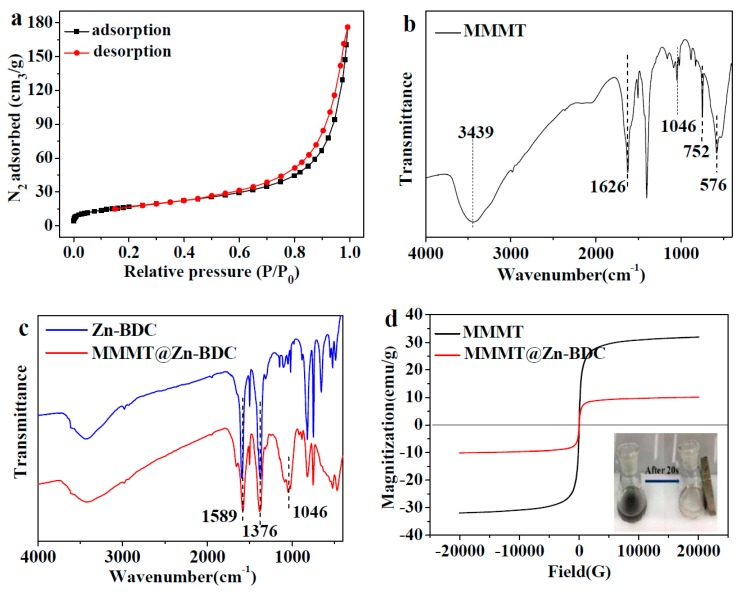
(**a**) N_2_ adsorption and desorption test for MMMT@Zn-BDC; FTIR spectra of (**b**) MMT, (**c**) Zn-BDC and MMMT@Zn-BDC, (**d**) VSM magnetization curves of MMMT and MMMT@Zn-BDC (inset: MMMT@Zn-BDC before and after application of a magnetic field for 20 s).

Figure 4a shows the results of N_2_ adsorption and desorption. The adsorption isotherm belongs to the type II isotherm. With increasing adsorption pressure, the first inflection point of the isotherm rapidly appears, indicating that monolayer adsorption is complete. As the adsorption pressure continues to increase, multi-layer adsorption gradually occurs, and when the saturated vapor pressure is reached, the adsorption layer is infinite. The surface area of MMMT@Zn-BDC was calculated to be 74.35 m^2^/g by the multipoint BET (Brunauer–Emmett–Teller) method [36], and the pore volume and pore size were 0.27 cm^3^/g and 15.48 nm, respectively.

#### 3.2.4. FTIR Analysis

Figure 4b is the FTIR spectra of MMMT. The peaks at 811 cm^−1^, 848 cm^−1^ and 1046 cm^−1^ belong to the asymmetric stretching of O–Si–O, deformation vibration of Al–OH–Mg, and stretching vibration of Si–O. These three characteristic peaks belong to MMT [37]. For MMMT prepared by the co-precipitation method, the stretching vibration of Fe–O caused a peak at 576 cm^−1^ [38], and the peak at 3439 cm^−1^ is the stretching vibration of O–H, which together show that MMMT was synthesized successfully. The band between 1376 cm^−1^ and 1589 cm^−1^ was attributed to the stretching vibration of the carbonyl group in BDC [39]. After Zn-BDC was coated onto the surface of MMMT, the characteristic absorption band remained, which shows that Zn-BDC was successfully synthesized on the surface of MMMT.

#### 3.2.5. VSM Analysis

The magnetic properties of MMMT and MMMT@Zn-BDC were studied by VSM analysis, and the results are shown in Figure 3d. The saturation magnetization of MMMT was 31.88 emu/g. After MMMT was coated onto Fe_3_O_4_, this value decreased to 10.09 emu/g. The decrease in magnetic properties indicated that Zn-BDC successfully covered MMMT. Good magnetic properties enable MMMT@Zn-BDC to accumulate rapidly under the external magnetic field. As shown in the inset of Figure 4d, MMMT@Zn-BDC could be separated by applying an external magnet for 20 s.

#### 3.2.6. XPS Analysis

**Figure 5:**
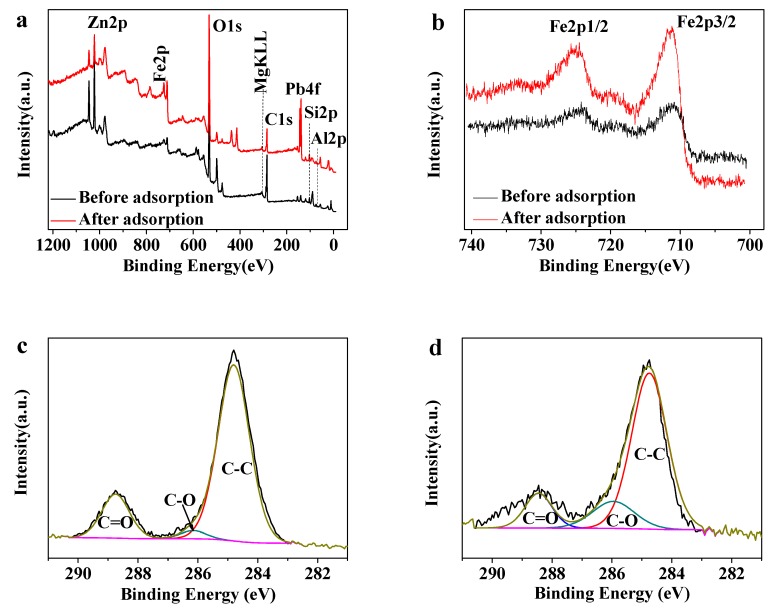
XPS spectra of MMMT@Zn-BDC: (**a**) wide scan, (**b**) Fe 2p; C 1s spectra of (**c**) MMMT@Zn-BDC and (**d**) MMMT@Zn-BDC loaded with Pb(II).

Figure 5a is the full XPS spectrum of MMMT@Zn-BDC before and after adsorption. The peak of Fe 2p belongs to Fe_3_O_4_, the peaks of Si 2p, Al 2p, Mg(KL_23_L_23_) originate from MMT [40], and the Zn 2p peak is from Zn-BDC. Meanwhile, the Pb4f peak appeared only after loading MMMT@Zn-BDC with Pb(II), demonstrating that MMMT@Zn-BDC is a good adsorbent for Pb(II). In Figure 5b, the peaks at 725.12 eV and 711.08 eV are characteristic of Fe 2p_1/2_ and Fe 2p_3/2_. Notably, the satellite peak of Fe_2_O_3_ at 714 eV did not appear in Figure 4b; this is because the peak is covered by the peaks of Fe 2p_1/2_ and Fe 2p_3/2_, which also proves Fe_3_O_4_ was successfully prepared [41]. In the C 1s spectrum in Figure 5c, the peaks at 288.75 eV, 286.14 eV and 284.80 eV belong to C=O, C–O and C–C, respectively [42]. However, as shown in Figure 5d, after loading MMMT@Zn-BDC with Pb(II), the above three peaks are moved to 288.45 eV, 285.96 eV, and 284.75 eV, respectively. The hydroxyl and carboxyl groups can complex with Pb(II), and caused the move of the binding energy.

### 3.3. Optimization of the Adsorption Conditions

#### 3.3.1. Effect of the pH of Pb(II) Solution

**Figure 6:**
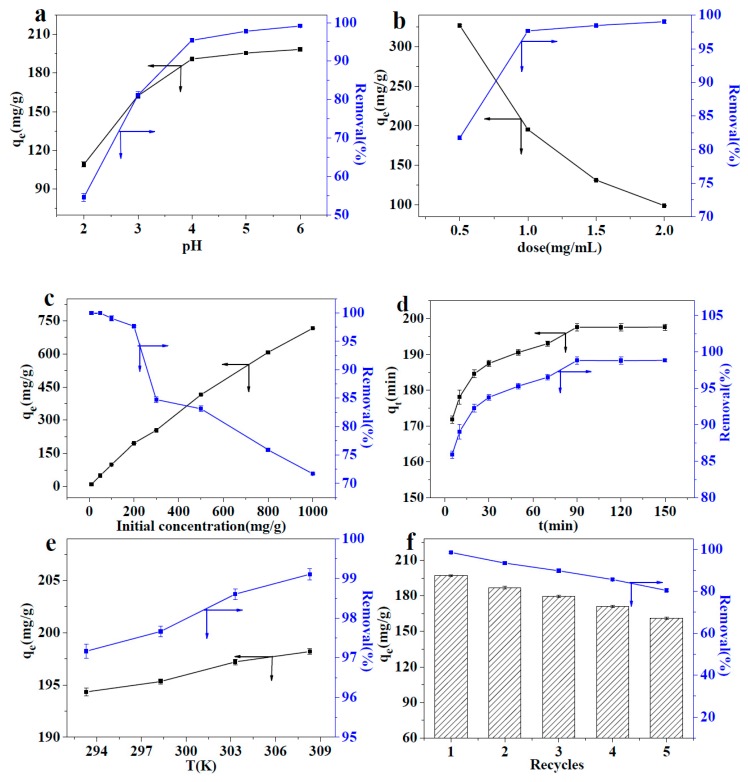
Effects of (**a**) pH, (**b**) adsorbent dosage, (**c**) initial concentration of Pb(II), (**d**) adsorption time, (**e**) adsorption temperature, and (**f**) recycling times on the adsorption capacity (left axis, black) and removal ratio (right axis, blue) of Pb(II) by MMMT@Zn-BDC.

A NaOH and HNO_3_ (0.1 mol/L) solution was used to adjust the pH of the Pb(II) solution from 2 to 6. When the pH is greater than 7, Pb(II) precipitates in the form of Pb(OH)_2_. Therefore, the pH was adjusted to 2−6 in this study. The effect of pH value on the adsorption capacity is shown in Figure 6a. MMMT@Zn-BDC (20 mg) was added to 20 mL Pb(II) with a concentration of 200 mg/L. The solution was agitated in a rocking bed. The adsorption capacity of MMMT@Zn-BDC to Pb(II) was then measured at each pH value. When the pH was between 2 and 4, the adsorption capacity increased rapidly with increasing pH. However, between 4 and 6, the adsorption capacity increased slowly. The pH of the Pb(II) solution can affect the surface charge of the adsorbent. At a lower pH, the surface negative charge of MMMT@Zn-BDC is relatively less; thus, its capacity to electrostatically adsorb Pb(II) is poor. With the increasing pH, the amount of surface negative charge of MMMT@Zn-BDC increases, which makes it favorable to positively charged Pb(II). This also proves that electrostatic adsorption is one way that the adsorbent adsorbs Pb(II). Because the initial pH of the 200 mg/L Pb(II) solution was 5.45, when the pH was not adjusted the adsorption capacity was 195.33 mg/g, which is close to that of the adsorption capacity with a pH of 6. So, there is no need to further adjust the pH.

#### 3.3.2. Effect of the Adsorbent Dose

Various amounts of MMMT@Zn-BDC (from 10 mg to 40 mg) were separately dispersed in 20 mL of Pb(II) solution with a concentration of 200 mg/L. Figure 6b shows the effect of the dosage of MMMT@Zn-BDC on the adsorption capacity. As the dosage of MMMT@Zn-BDC increased from 10 mg to 40 mg, *q*_e_ decreased from 326.99 to 99.03 mg/g. At the same time, the removal ratio increased from 81.74% to 99.03%. When the dosage of the adsorbent was low, the amount of Pb(II) was excessive for the available adsorption sites. At this time, the probability that adsorption sites were occupied was high, so the adsorption capacity was high. When the dosage of the adsorbent increased, sufficient adsorption sites were available for Pb(II), so the removal ratio increased while *q*_e_ decreased because of a large number of unoccupied adsorption sites exist in the adsorbent. When the adsorbent dosage was 1.0 mg/mL, the removal ratio reached 97.67%. As the adsorbent dosage increased, the change in the removal ratio was not obvious. Thus, considering the adsorption capacity, we chose 1 mg/ml as the dosage of the adsorbent for further experiments.

#### 3.3.3. Effect of the Pb(II) Concentration

The initial concentration of Pb(II) can have serious implications for the adsorption capacity of MMMT@Zn-BDC. Therefore, we investigated the adsorption capacity of the solution with concentrations ranging from 10 to 1000 mg/L. Similarly, 20 mg of MMMT@Zn-BDC was separately dispersed in 20 mL different concentrations of Pb(II) solutions, the results are shown in Figure 6c. Even if the concentration reached 1000 mg/L, the adsorption was still not saturated but the removal rate decreased to 71.7% at this concentration. In practical applications, the removal ratio is an important factor for evaluating the adsorption properties of an adsorbent, so we did not continue to increase the Pb(II) concentration. With the increase in Pb(II) concentration from 10 to 1000 mg/L, the adsorption capacity increased from 10 mg/g to 717.01 mg/g. This is because at a higher concentration, excessive Pb(II) is available for the adsorption sites of MMMT@Zn-BDC, thus, it is easy to capture Pb(II) for the adsorbent sites. When the concentration of Pb(II) was below 200 mg/L, the lead ions were almost completely removed, and the removal ratio reached 97.67%. So, the Pb(II) concentration of 200 mg/L was chosen as the experimental concentration for further experiments.

#### 3.3.4. Effect of Adsorption Time

The relationship between adsorption time and adsorption capacity was also investigated in this study. MMMT@Zn-BDC (20 mg) was dispersed in 20 mL of Pb(II) solution, and the concentration remained at 200 mg/L. The adsorption capacities at different times are shown in Figure 6d. During the primary stage of the adsorption process, the increase in the adsorption capacity is an almost logarithmic relationship. As adsorption proceeded between 0 and 20min, a large number of free adsorption sites could rapidly bind Pb(II). As the adsorption time was prolonged, the adsorption sites gradually become occupied by Pb(II), and the increase in the adsorption capacity started to slow down. After 90 min of the adsorption process, the adsorption capacity was 195.33 mg/g, and the removal rate was 97.67%. Beyond this adsorption time, the adsorption capacity remained unchanged. Thus, the time that the adsorption reached equilibrium was 90min, at the concentration of 200 mg/L.

#### 3.3.5. Effect of the Adsorption Temperature

The adsorption capacity of MMMT@Zn-BDC for Pb(II) was measured from 293.2 K to 308.2 K. The dosage of the addition of MMMT@Zn-BDC in Pb(II) solution was 1 mg/mL. The effects of temperature on the adsorption capacity are shown in Figure 6e. With increasing temperature, the adsorption capacity increased from 194.34 mg/g to 198.21 mg/g. At the same time, the removal rate increased from 97.17% to 99.11%. The results show that the adsorption process of MMMT@Zn-BDC for Pb(II) is an endothermic process. Therefore, increasing the temperature is beneficial to the adsorption process.

#### 3.3.6. Effect of the Recycling Process

Dilute HCl (0.1 mol/L) was used to desorb the Pb(II) loaded on the adsorbent and after detachment the adsorbent was repeatedly applied to remove Pb(II). The effects of reuse cycles on adsorption capacity and removal ratio are shown in Figure 6f. With the increasing number of reuses, the adsorption capacity decreased gradually. After being reused five times, the adsorption capacity was 160.90 mg/g, that is, the adsorption capacity declined. However, the adsorption rate remained at 80.45%, which shows that MMMT@Zn-BDC has good recyclability.

### 3.4. Adsorption Mechanism

The adsorption isotherm was determined by the Langmuir and Freundlich models. The adsorption data were taken from Figure 5c. In general, the Langmuir isotherm model usually assumes that the adsorption capacity of the adsorption site is uniform, and this isotherm model is often applied to describe monolayer adsorption processes. On the other hand, the Freundlich isotherm model is used to describe the effect of the adsorbate concentration on the adsorption capacity and whether adsorption is prone to occur. The two equations of the adsorption isotherms are as follows:

(5) $$\mathrm{Langmuir}\;\mathrm{equation}:\;\frac{C_{e}}{q_{e}}=\frac{1}{bq_{m}}+\frac{C_{e}}{q_{m}}$$

(6) $$\mathrm{Freundlich}\;\mathrm{equation}:\;\mathrm{lg}q_{e}=\mathrm{lg}K_{F}+\frac{1}{n}\mathrm{lg}C_{e}$$

where the maximum adsorption capacity (*q*_m_, mg/g) can be obtained from the Langmuir model, *b* and *n* are the constants of Langmuir and Freundlich isotherm models, respectively, and *K*_F_ is a constant representing the adsorption capacity. *R*_L_ is a dimensionless constant for Langmuir isotherm model, and it is used to determine whether the adsorption behavior is preferential adsorption. The equation is as follows:

(7) $$R_{L}=\frac{1}{1+bC_{0}}$$

**Figure 7:**
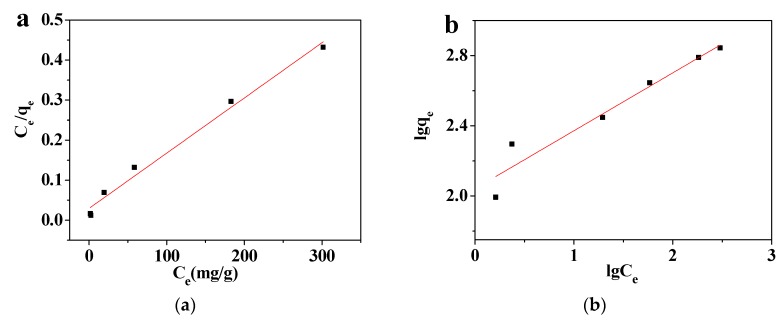

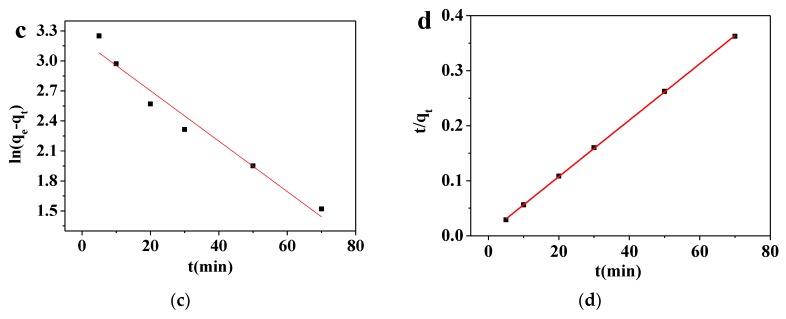
Fitting curves of the Pb(II) adsorption process by MMMT@Zn-BDC: (**a**) the Langmuir and (**b**) the Freundlich isotherm models, (**c**) a pseudo-first order and (**d**) a pseudo-second order kinetic models.

Figure 7a,b shows the curves fitted by the Langmuir and Freundlich isotherm models, Table 2 shows all of the related parameters while the correlation coefficients (*R^2^*) fitted by the Langmuir and Freundlich isotherm models are 0.9850 and 0.9239, respectively. According to its higher correlation coefficient, the adsorption process of MMMT@Zn-BDC for Pb(II) is better described by the Langmuir isotherm model, which indicates that Pb ions do not interact. The adsorption capacity of each site on the adsorbent is the same, and monolayer adsorption accords with the adsorption process. Moreover, *q*_m_ reached 724.64 mg/g. In Table 3, the calculated *R*_L_ values are all less than 1, therefore, it can be concluded that this adsorption process is favorable [43].

Diffusion is the main adsorption mode for adsorption processes conforming to a pseudo-first-order kinetics model. The adsorption process that accords with this model, only have one kind of binding site while the adsorption process that accords with the pseudo-second-order model is chemical adsorption, which involves the sharing or transfer of electron pairs between the adsorbent and the adsorbate.

(8) $$\mathrm{Pseudo}-\mathrm{first}-\mathrm{order}\;\mathrm{equation}:\;\ln(q_{e}-q_{t})=\ln q_{e}-k_{1}t$$

(9) $$\mathrm{Pseudo}-\sec\mathrm{ond}-\mathrm{order}\;\mathrm{equation}:\;\frac{t}{q_{t}}=\frac{1}{k_{2}q_{e}^{2}}+\frac{t}{q_{e}}$$

*k*_1_ (1/min) and *k*_2_ (g/mg∙min) are the constants in these two models. The linear equation fitted by the above two models is shown in Figure 7c,d, and the parameters were calculated and are shown in Table 4. The correlation coefficient (*R*^2^) of the pseudo-second-order kinetics model is 0.9999, and the *q*_e,cal_ is 194.93 mg/g; this result is similar to the experimental result (195.33 mg/g). Meanwhile, the *R*^2^ of the pseudo-first-order kinetics model is 0.9566, and the *q*_e,cal_ is 8.71.Therefore, we can conclude that the adsorption process of MMMT@Zn-BDC on Pb(II) fits the pseudo-second-order kinetics model, combined with the hypothetical premise of the pseudo-first-order kinetics, the adsorption process of MMMT@Zn-BDC for Pb(II) is chemical adsorption [44].

Moreover, the adsorption capacity changes in the range of 293.2 K to 308.2 K were also investigated. These data were used to study the adsorption thermodynamics, and the relevant parameters were calculated by Equation (10):

(10) $$\Delta G=-RT\ln\frac{q_{e}}{C_{e}}=-RT(-\frac{\Delta H}{RT}+\frac{\Delta S}{R})$$

where Δ*G* (kJ/mol), Δ*H* (kJ/mol) and Δ*S* (kJ/mol) represent Gibbs free energy, enthalpy and entropy, respectively. *T* (K) is the thermodynamic temperature, and *R* is the gas constant. Table 5 shows the calculated results.

The negative Δ*G* indicates that the adsorption process of MMMT@Zn-BDC for Pb(II) is endothermic and spontaneous under constant pressure. Meanwhile, Δ*H* is greater than 40 kJ/mol, indicating that the adsorption process is chemisorption. This conclusion is in agreement with the adsorption kinetics model. Therefore, a higher temperature is beneficial to the adsorption process.

**Figure 8:**
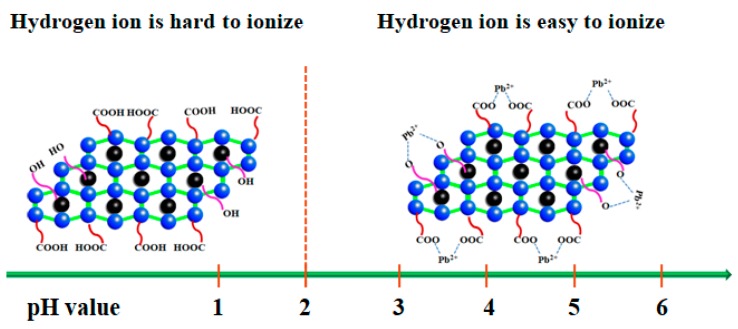
Diagram of the adsorption mechanism.

In general, based on the results of the optimization process of the adsorption conditions, the adsorption capacity of MMMT@Zn-BDC for Pb(II) suffers most with changes in the pH. The hydroxyl and carboxyl groups of the adsorbent surface are not easily dissociated when the pH is lower than 2. Moreover, a high concentration of hydrogen ions is not conducive to ion exchange between MMMT and Pb(II). At a higher pH, hydroxyl and carboxyl groups are easier to dissociate, and these dissociated hydroxyl and carboxyl groups easily form complexes with Pb(II). The study of adsorption kinetics and adsorption thermodynamics confirmed that the adsorption process is chemisorption, as shown schematically at different pH values in Figure 8.

In this study, MMMT@Zn-BDC is a type of composite material. In order to further understand the advantages of MMMT@Zn-BDC for the adsorption of Pb(II), we compared MMMT@Zn-BDC with the *q*_m_ and equilibrium time of the adsorption of several reported adsorbents (Table 6).

## 4. Conclusions

For the first time, we prepared a magnetic composite material by combining Fe_3_O_4_, MMT and Zn-BDC using a co-precipitation method and in situ polymerization. This magnetic compound was applied to adsorb Pb(II). Based on the results of adsorption isotherm and adsorption kinetics, the adsorption behavior is in accordance with the Langmuir isotherm model and pseudo-second-order kinetics model, and the process of MMMT@Zn-BDC adsorbing Pb(II) is endothermic. The time required to reach adsorption equilibrium is 90 min. In addition, MMMT@Zn-BDC also exhibited excellent recycling performance. The removal rate remained at 80.45% after recycling five times. Its easy separation and sustainability make MMMT@Zn-BDC a superior adsorbent for removing Pb(II).

## Figures and Tables

**Table 1:** The effect of the addition of Fe_3_O_4_, MMT, and Zn-BDC on the adsorption capacity.

Fe_3_O_4_:MMT (g/g)	BR (%)	*q*_e_ (mg/g)	MMMT: Zn-BDC (g/g)	BR (%)	*q*_e_ (mg/g)
2:1	97.26 ± 0.32	71.36 ± 0.23	2:1	69.12 ± 0.28	224.66 ± 0.18
1:1	96.83 ± 0.41	98.67 ± 0.19	1:1	66.39 ± 0.57	280.51 ± 0.23
2:3	65.94 ± 0.29	99.06 ± 0.22	2:3	32.94 ± 0.69	281.48 ± 0.35
1:2	49.61 ± 0.30	98.92 ± 0.47	1:2	19.97 ± 0.64	282.04 ± 0.44

**Table 2:** Langmuir and Freundlich models’ parameters for removing Pb(II) with MMMT@Zn-BDC.

*T* (K)	Langmuir Isotherm			Freundlich Isotherm		
	*q*_m_ (mg/g)	*b* (L/mg)	*R^2^*	*K*_F_	*n*	*R^2^*
298.2	724.64	0.047	0.9850	110.07	3.03	0.9239

**Table 3:** *R_L_* for Pb(II) removal by MMMT@Zn-BDC.

***C*_0_** **(mg/L)**	10	50	100	200	300	500	800	1000
***R*_L_**	0.68	0.30	0.18	0.096	0.066	0.041	0.026	0.021

**Table 4:** The kinetic models’ parameters of the Pb(II) adsorption process by MMMT@Zn-BDC.

*q*_e,exp_	Pseudo-First-Order Model			Pseudo-Second-Order Model		
	*q*_e,cal_	*k*_1_	*R* ^2^	*q*_e,cal_	*k*_2_	*R* ^2^
197.63	8.71	0.02390	0.9566	194.93	0.02521	0.9999

**Table 5:** The parameters of thermodynamics for the Pb(II) adsorption process on MMMT@Zn-BDC.

**Δ *G* (kJ/mol)** ***T* (K)**				**Δ *H*** **(kJ/mol)**	**Δ *S*** **(J/mol∙K)**
293.2	298.2	303.2	308.2		
−8.62	−9.26	−10.74	−12.06	60.779	0.2359

**Table 6:** Comparison of the adsorption capacity of MMMT@Zn-BDC for Pb(II) with that of other composite materials.

Sorbent	*q*_m_ (mg/g)	Adsorption Time	Ref.
AS-ACI	133.3	150	[45]
Mg_2_Al–CO_3_–LDH	123	120	[46]
m-CS/PVA/CCNFs	175.4	200	[47]
Zr-MOF	166.74	120	[48]
MIL-101-NH_2_@SA	529.10	180	[49]
MMMT@Zn-BDC	724.64	90	This work
